# The Training Complex: Shattering of the *Coniunctio*, Boundary Violation, and Sibling Turbulence on the Horizontal Plane

**DOI:** 10.1111/1468-5922.70076

**Published:** 2026-07-30

**Authors:** Anne Marie Allen

**Affiliations:** ^1^ West Sussex UK

**Keywords:** alchemical *vas*, analytic siblings, ethical boundary violation, opposites, shattering of the *coniunctio*, training complex, turbulence on the horizontal plane, vas alchimique, turbulence sur le plan horizontal, violation éthique des limites, frères et sœurs analytiques, fragmentation de la coniunctio, complexe de formation, opposés, alchemistisches *vas*, Turbulenzen auf der horizontalen Ebene, Verletzung ethischer Grenzen, analytische Geschwister, Zerbrechen der *coniunctio*, Ausbildungskomplex, Gegensätze, : *vaso alchemico*, turbulenze sul piano dell'esperienza, violazioni dei confini etici, fratellanza analitica, distruzione della *coniunctio*, complesso del training, opposti, : алхимический сосуд, турбулентность в горизонтальной плоскости, этическое нарушение границ, аналитики‐сиблинги, разрушение *coniunctio*, тренинговый комплекс, противоположности, : *vas* alquímico, turbulencia en el plano horizontal, violación de los límites éticos, hermanos analíticos, ruptura de la *coniunctio*, complejo de formación, opuestos, 炼金术*容器*, 水平面上的动荡, 伦理边界侵犯, 分析中的手足, *整合*的破碎, 培训情结, 对立面

## Abstract

This paper explores what is happening, for a trainee, when an ethical boundary violation occurs by the analyst with a fellow peer in training. I am approaching this through the Jungian concept of the alchemical *vas* as I attempt to make meaning of a catastrophic collapse of the analytic work. The boundary edges have shifted, catapulting the analyst and patient—from something reliable—into an altogether darker and more inexplicable experience. There has been a trespass, and something has been stolen. The author explores how the destructive forces at play are reminiscent of the envy and hatred located in sibling dynamics. Furthermore, the internal chaos is also about how trainees may experience their “sibling” peers in everyday ways. The author describes these as chaotic forces and what unfolds within a training organization, as it responds and deals with subsequent reverberations, “The Training Complex”, suggesting that the symbolic representation of siblings in this complex is that of opposites.

## Abstract (fr)

Cet article explore ce qui se produit, pour un analyste en formation, lorsqu’une violation éthique des limites est commise de la part de l’analyste avec un pair également en formation. L’approche s’appuie sur le concept jungien du vas alchimique afin de tenter de donner sens à un effondrement catastrophique du travail analytique. Les frontières ont été déplacées, propulsant analyste et patient—d’un espace auparavant fiable—dans une expérience beaucoup plus sombre et difficile à comprendre. Il y a eu transgression, et quelque chose a été dérobé. L'auteur examine en quoi les forces destructrices à l'œuvre rappellent la jalousie et la haine qui caractérisent les relations entre frères et sœurs. De plus, le chaos interne concerne aussi la manière dont les analystes en formation peuvent faire l’expérience de leurs pairs « fraternels » dans la vie quotidienne. L’auteure décrit ces phénomènes comme des forces chaotiques et analyse ce qui se déploie au sein d’une organisation de formation lorsqu’elle réagit et fait face aux répercussions ultérieures—le « complexe de formation »—suggérant que la représentation symbolique des frères et sœurs dans ce complexe est celle des opposés.

## Abstract (de)

Diese Arbeit untersucht, was mit einem Ausbildungskandidaten geschieht, wenn ein Analytiker gegenüber einem anderen Ausbildungskandidaten eine ethische Grenze überschreitet. Ich nähere mich diesem Thema anhand des Jungianischen Konzepts des alchemistischen *vas*, um im katastrophalen Scheitern der analytischen Arbeit einen Sinn zu finden. Die Grenzen haben sich verschoben und den Analytiker und den Patienten – aus einer verläßlichen Position – in eine völlig düstere und unerklärliche Erfahrung katapultiert. Es gab einen Übergriff und etwas wurde gestohlen. Die Autorin untersucht, wie die destruktiven Kräfte, die hier wirken, an den Neid und Haß in Geschwisterbeziehungen erinnern. Darüber hinaus geht es bei dem internen Chaos auch darum, wie Ausbildungskandidaten ihre "Geschwisterkollegen" im Alltag erleben. Der Autor beschreibt diese als chaotische Kräfte und die daraus resultierenden Reaktionen und Auswirkungen innerhalb einer Ausbildungsorganisation – den sogenannten "Ausbildungskomplex". Dies deutet darauf hin, daß die Geschwister in diesem Komplex symbolisch für Gegensätze stehen.

## Abstract (it)

L'articolo esplora cosa accade ad un allievo in training quando si verifica una violazione dei confini, da parte dell'analista, con un collega di training. Cercando di dare significato alla caduta rovinosa del lavoro analitico, rifletterò su questo tema utilizzando l'idea junghiana dell'*alchemical vas*. Una volta che i confini sono stati spostati, catapultando analista e paziente ‐da qualcosa di concreto‐ dentro qualcos'altro, un'esperienza ben più oscura e inspiegabile. C'é stato uno sconfinamento, e qualcosa è andato perso. L'autore esplora come le forze distruttive che sono in campo siano una reminiscenza dell'invidia e dell'odio che erano nelle dinamiche tra fratelli. Inoltre, il caos iniziale può anche riferirsi a come gli allievi in training fanno esperienza dei loro “fratelli” allievi, nella quotidianità. L'autore descrive queste forze come caotiche e descrive come si presentano anche nell'organizzazione del training, e come questo abbia le sue riverberazioni, “Il Complesso del Training”, suggerisce che la rappresentazione simbolica dei fratelli si presenti attraverso l'opposto.

## Abstract (ru)

В статье исследуется реакция кандидата на этическое нарушение границ между его аналитиком и его коллегой по тренингу. Я подхожу к этой ситуации через призму юнгианской концепции алхимического сосуда и пытаюсь осмыслить ее пагубные последствия для аналитической работы. При смещении границ аналитик и пациент вместо надежных рамок оказываются в ситуации более смутной и менее понятной. Происходит незаконное нарушение границ: нечто было похищено. Автор исследует разворачивающееся действие деструктивных сил как аналог зависти и ненависти в сиблинговых отношениях. Внутренний хаос также вызван тем, как кандидаты могут воспринимать своих «сиблингов» в повседневной жизни. Автор описывает такое нарушение границ как действие хаотических сил, а реакцию на это и последующие отклики называет «тренинговым комплексом», предполагая, что сиблинги символическая представлены в этом комплексе как противоположности.

## Abstract (es)

Este artículo explora lo que le sucede a un candidato, cuando el analista comete una violación de los límites éticos con un compañero de formación. Abordo este tema a través del concepto Junguiano del “vas alquímico”, en un intento por dar sentido a un colapso catastrófico del trabajo analítico. Los límites se han desplazado, catapultando a analista y paciente —desde algo confiable— hacia una experiencia tanto más oscura como inexplicable. Ha habido una transgresión, y algo ha sido robado. El autor explora cómo las fuerzas destructivas en juego son reminiscencias de la envidia y el odio presentes en la dinámica entre hermanos. Además, el caos interno también se refiere a cómo los candidatos pueden experimentar a sus compañeros “hermanos” en los modos cotidianos. El autor describe estas como fuerzas caóticas y como aquello que se desarrolla dentro de una institución de formación, a medida que responde y lidia con las subsiguientes reverberaciones, “El Complejo de Formación”, sugiriendo que la representación simbólica de los hermanos en este complejo es la de los opuestos.

## Abstract (zh)

本文探讨了, 当分析师和受训者的同伴一起, 侵犯伦理边界时, 受训者所经历的状况。我将通过荣格的炼金*术“容器”*概念来解读这一现象, 试图阐释分析工作遭遇灾难性崩溃的深层含义。边界线已然位移, 将分析师与患者从原本可靠的状态, 猛然抛入一种黑暗且难以名状的体验之中。边界已被逾越, 某种东西已被窃取。 作者探讨了其中起作用的破坏性力量如何令人联想到手足关系中的嫉妒与仇恨。此外, 这种内在的混乱也关乎受训者如何在日常互动中体验其“手足”般的同伴。作者将这些描述为“培训情结”, 作者描述了这一情结中, 混沌的力量, 以及培训组织在应对后续余波时所发生的情形, 暗示该情结中手足关系的象征性表现是带有对立面性质的。

## Introduction

My paper is stimulated by a personal experience of my analysis ending abruptly as the analyst was suspended, pending a ‘Fitness to Practise’ investigation, due to a relationship with a patient. The experience happened towards the end of my training, in what had felt like a difficult analysis, yet it was devastating and complex as the shock of his being suspended for being in a relationship with a patient turned to incomprehension, derealisation, and feelings of deception and betrayal at the realisation it was with a colleague (a fellow ‘sibling’) with whom I was training.

I observed myself moving back and forth between a place of strength and one of futility and I find myself reflecting on how one can ever come to terms with such a catastrophic failure. I come to this over many months now having thought about container and containment in analysis and wanting to understand what happens when the container breaks. I have thought of vessels and wombs and breasts—of things that contain—of the frame and therapeutic relationship and holding.

I have lived the shattering of the *coniunctio* between analyst and patient, yet it is not like a thought or a memory, more like a piercing cry, or a stamping sensation on metal, or something incongruous and terribly hard to say unless it is done in poetry. It is difficult to piece together what it was like to have the experience of being in the analytic container and how the relationship broke down in a moment, became irretrievable, lost, broken beyond repair. It is hard to grasp a narrative that goes round and round, nonsensical and occasionally sensical, but distressing events will do that.

Many papers have attempted to grasp aspects of this narrative, to make sense of the nonsensical, and these contributions allow theoretical understanding and create meaning: a vital response to a fragmented reality. Jean Kirsch’s personal reflection on John Perry’s “incomprehensible blindness” (Kirsch, 2021, p. 223) tells of her attempts to understand his perplexing state. “I believe that he suffered a profound and chronic ego‐possession by his countertransference fantasies, which seemed to form a kind of walled‐off and autistic entity under the banner of ‘love’” (Kirsch, 2021, p. 223). I think of her writing these words as an attempt to contain and transform her own experience. Glen Gabbard has written tirelessly on the impact of sexual boundary violation in the analysis and within the training organisation, yet his 30‐year retrospective records a sense of “growing pessimism” (2017, p. 151) on turning the tide of analyst–patient relationships and refers to the “pervasive self‐deception of analysts and therapists” (2017, p. 151). It can be disheartening to see how little changes.

Qualitative data analysis within one institute recognised the substantial personal toll on members after two senior members were found to have committed ethical boundary violations: “I felt downtrodden and crushed inside” (Burka et al., 2019, p. 256). The repercussions were widespread when thinking about confidentiality: “I had no recourse, nowhere to go to make sense of it. Just bits and pieces, fragments here and there, floating in orbit” (Burka et al., 2019, p. 258). It is in fact difficult to separate the individual from the group when it comes to an ethical boundary violation as: “The damage that spread throughout the institute, demonstrating that a violation of one is a violation of many” (Burka et al., 2019, p. 247). This element of contagion is reflected in the journey of this paper as the shattering of the *coniunctio* between analyst and patient—as a result of boundary violation with a peer sibling—became about more than the impact on the breakdown of an analysis, but of a more widespread disturbance that affected many individuals within the training community.

## Alchemical *Vas*—Where the Transformation Takes Place

The first illustration of the *Rosarium Philosophorum* is of *The Mercurial Fountain* and depicts the alchemical container as described by Jung as “the basin below … the *vas Hermeticum*, where the transformation takes place” (1946, para. 402). He also makes links to “chaos” and separately the vessel as “uterus” (1946, para. 402) and so I hear a suggestion of how this process can be both chaotic and yet creative. The infant’s birth from the rounded womb container is our first experience falling from the *uroboros* into life. The alchemical *vas* becomes a place of transformation in the work between analyst and patient and one where Stanton Marlan conjectures, “the matters of psyche could be heated up, cooked, coagulated, distilled and transformed” (Marlan, 2006, p. 289).

This powerful process is picked up by Andrew Samuels in considering how the *coniunctio* “refers metaphorically to the deep and pervasive intermingling of the two personalities involved in therapy” (Samuels, 2006, p. 186). The analytic experience is expected to be one of psychic upheaval, of confronting shadow aspects whilst being with, and in dialogue with, an other. The process is perilous for the analyst and trainee as the unconscious elements mix together. It is critical for the analyst to have experience of working within the shadow complex, or any overpowering and unconscious need to merge (that overlaps with the trainee’s own unmet needs) could undo the creative tension of fusion and separation held within the work. The theoretical womb‐*vas* must hold these unconscious aspects.

It is not a container in the sense of Bion’s container contained when a mother is able to respond to and contain her infant’s dread through her sense of reverie; neither is the *vas* like Winnicott’s safe place of holding, with a mother providing space and time for her infant. The Great Mother can get inside this alchemical vessel, devouring her child in a flash. It can be painstaking and painful holding the blood and the gore. This container sits on the edge of reason, but it has depth and purpose. The journey will unfold, maybe not so methodically as within the *Rosarium* woodcuts, yet it is an enduring space for two people to come to know themselves better.

Experiences of boundary violation within training risk weakening the capacity of the training womb‐*vas*, for the peers that share the analyst, that might result in internal chaos. It may become hard to differentiate between what the patient is bringing and what the analyst is caught up in—whether the blood and gore is the right sort of blood and gore in fact.

The sixth *Rosarium* illustration showing Death (see Figure 1): “Here King and Queen are lying dead/In great distress the soul is sped” (Jung, 1946, para. 467) is where the experienced analyst must use every resource to steer the way, entwined as they are in a psychological twinning with the patient. The process is an expected part of an analysis in that no one can make a psychological descent without moving into shadow aspects of themselves. “No new life can arise, says the alchemists, without the death of the old” (1946, para. 467).

**Figure 1:**
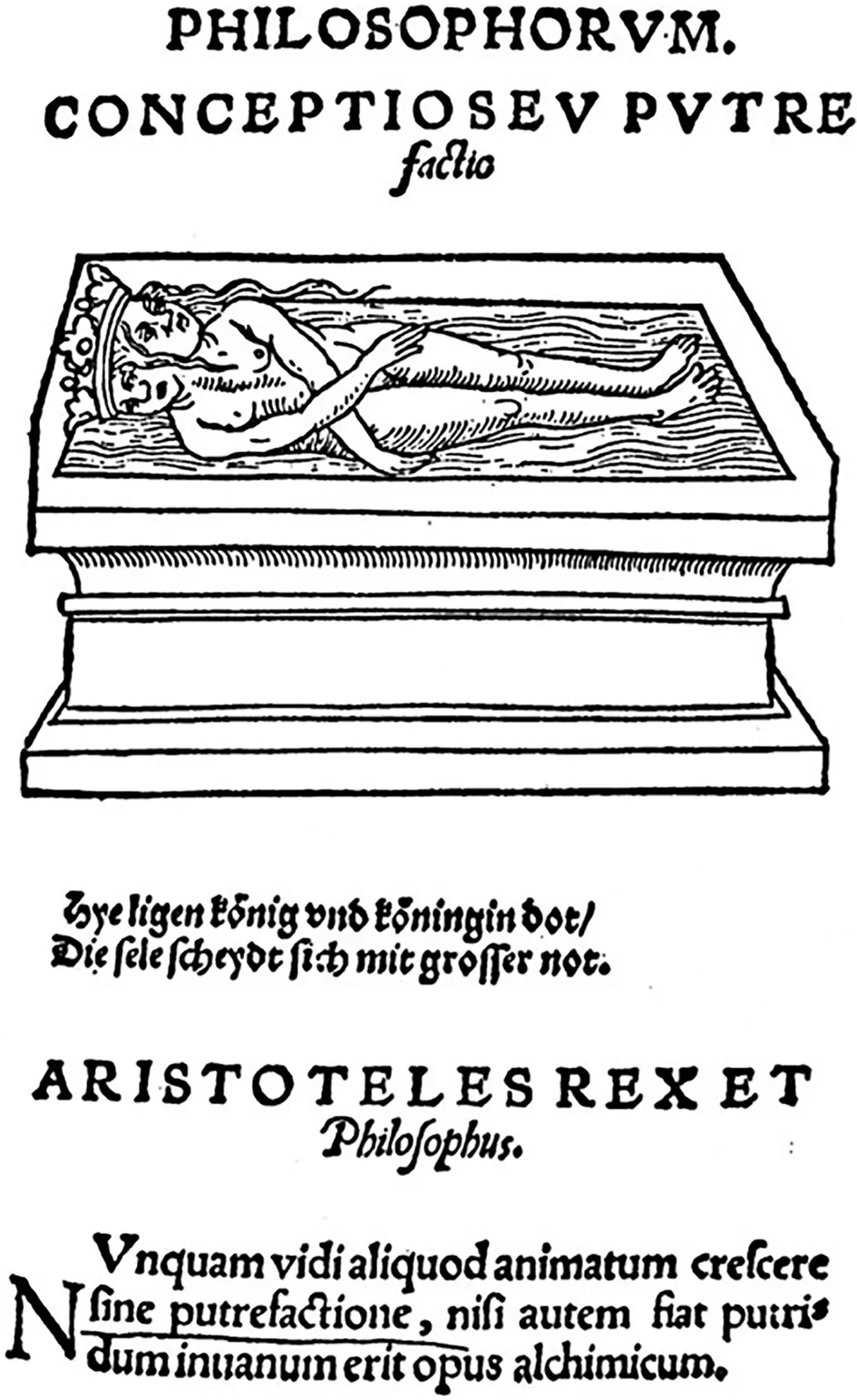
*Rosarium philosophorum*: “Death.” *Source:* In C. G. Jung (1946/1966), *The Psychology of the Transference*, *Collected Works*, Vol. 16 (Princeton, NJ: Princeton University Press), ¶468 (plate). Reproduced with permission of Princeton University Press.

I suggest ‘death’ is the place where work is more likely to go awry, where one loses sight of the bigger picture, amidst extremely powerful and unknown forces. Here it is hard to hold onto aspects of self and other, and I am reminded of Erich Neumann’s description: “Over against all this the ego–consciousness, the individual–remains small and impotent. It feels itself a tiny, defenseless speck, enveloped and helplessly dependent, a little island floating on the vast expanse of the primal ocean” (Neumann, 1954/2014, p. 40). This is the primitive ego holding on for dear life in the work.

Jung says: “The reckoning is presented, and a dark abyss yawns. Death means the total extinction of consciousness and the complete stagnation of psychic life” (1946, para. 469). This place links to the analyst who commits an ethical boundary violation and who is psychologically entwined with trainees who must endure the loss of the relationship, and the entire psychological process held within the analytic *vas*.

The alchemical *vas* is a place of transformation because it acknowledges the darkness and what can be the tormented nature of relationship: “*nigredo*—the blackness, the terrible depression and state of dissolution” (von Franz, 1980, p. 220) or “*nigredo*—suicide, madness, and self‐castration” (Schwartz‐Salant, 1998, p. 142). It is also a place full of shards and wreckage. The Attis–Cybele myth represents these extreme events through the relationship of Cybele, the Great Mother, and Attis her son and lover. Here the disturbing fusion carries themes of “deep love and jealousy but also …. revenge, betrayal, and madness.” (Schwartz‐Salant, 1998, p. 125). I see parallels in these extreme aspects of the myth with the experience of taboo and intensity of the impact of ethical boundary violations. I cannot help thinking that these themes coalesce with those of a trainee impacted by an ethical boundary violation, as well as the analyst committing an act of professional sabotage.

One of the most striking aspects is the self‐destructiveness in the analyst’s behaviour that is obvious to everyone but the analyst. It appears that the analyst unconsciously enacts a masochistic scenario that relates to childhood wishes of self‐sacrifice. Often, the details of this fantasy involve a wish to “go out in a blaze of glory” by acting on incestuous wishes for a parent and experiencing the retaliation and punishment for a forbidden act of pleasure. (Gabbard, 2016, pp. 21–22)

## Alchemical *Vas*—Stuck in *Nigredo*

It can be difficult to fully understand the psychic destructiveness of the experience for all concerned. For the trainee who hears there has been a boundary violation with a patient, there will be a huge and jarring shock, and yet despite the devastation and the grief, there may be an initial response that is to try and cling to the relationship with the analyst—to the familiar structure of sessions in the week, to the concreteness, the routine, the place—as well as the person of the analyst.

I suggest there is a second break in the container upon hearing that the breach occurred with a known peer, and that this break will cause the greatest psychic discomfort. This is certainly a further violation of the work. The emotional response will move into something bigger, murkier, more troublesome. I think this is moving from the grief of the vertical container break into the smithereens of the horizontal container break. From death to destruction. From grief to betrayal. This is the moment when a life‐changing truth shatters the reality of the enduring vessel. Concrete external reality overtakes the symbolic internal world.

I remember falling through reality in that moment, falling into a new and devouring truth. It is like the baby’s passage from movement to not movement and the awfulness of stopping when the newly born infant comes into contact with the ground for the first time: “Nothing moves. The world has frozen, died. It is the unknown” (Le Boyer, 1974/1976, p. 94) or “of being unbounded, de‐wombed” (Boadella, 1987, p. 57). These prosaic yet moving descriptions resonate with the experiences of trainees whose analyst has ‘put them down’, ‘miscarried’ them suddenly. There are connections to be made when thinking of the impact of boundary violation with early experiences: pre‐birth, natal, infant. As a clinician, it reminds me of the responsibility we carry in holding these young and vulnerable parts.

Much can be learned from how a person is impacted. The personal experience of every trainee who has fallen out of therapy in this way may reveal something more about the relationship inside the container. How the bough breaks is unique to the relationship, like a personal fault line cracking apart under the strain of enactment. The idea of being in a transformational process becomes problematic because the analytic process has not been able to reach a desired outcome or mutual ending. I am thinking that when containers break there are smithereens, shards, pieces of wreckage that cannot come back together as a whole.

I think it is the taboo of a boundary violation that makes the break in the container feel so dangerous (Burka et al., 2019; Keenan, 1995). The therapeutic relationship is unique and incomparable to other types of relationship and there are reasons why the taboo is necessary. To break this code is a betrayal of what the trainee can reasonably expect from an analysis. Usually, the taboo of an ethical breach will result in being struck off for misconduct. Here is the bloodshed and violence as the analyst is castrated from the analytic relationship and then, as a result, is cast out from the training organisation, severed from patients, peers and the containment of the profession. The trainee must find a way to absorb the new reality whilst attempting to remain within an analytical process. To understand this drama is to begin to understand the universal shadow theme of Cybele‐Attis, and to understand how we are left to wrestle with the same unnerving themes in our contemporary alchemical narrative, as the alchemists have done for thousands of years with the *prima materia*.

The Cybele–Attis myth is a narrative that spans centuries, cultures. It is the single story that becomes many stories that *became* the mythical story and reaches into the heart of how the narrative of the ethical boundary violation becomes woven into psychoanalytic history, organisational history, trainee history, and how we deal with the violation. ‘It has always happened’, ‘it will go on happening’, ‘it will happen again’ are the very real responses to the story. These implications suggest acceptance, powerlessness, the impotence of the ‘defenseless speck’. There are those who cannot recognize the pain. I have wondered if a murdered trainee would provoke the same lack of action in us. Something here gets castrated in the rather ordinary response as if suddenly symbolism is no longer important, when symbolism is everything. As Jung suggested, “It should never be forgotten that it is a *symbolical* relationship whose goal is complete individuation” (Jung, 1946, para. 261). The destruction of the symbolic relationship becomes a desecration of the internal sacred spaces, the profound and vulnerable depths to which the work has descended. This is perhaps the greatest loss. Jung’s own boundary violation in relation to Sabina Spielrein shows how the shadow intrudes on symbolic and clinical intention. Her diary and letters capture vividly the emotional pain and upheaval Spielrein experienced through Jung’s deception (Carotenuto, 1980/1984).

There is also something particularly destructive when the task is not completed, when the work is abandoned, when the decision to end is removed from the patient. A planned ending is a necessary and valuable function of the therapeutic relationship, and a catastrophic ending deprives analyst and patient of something meaningful. Kenneth Frank states, “No matter what has come before, if the ending of the analytic relationship is not managed sensitively, much good may be undone” (Frank, 2009, p. 136). This loss of an ending or a “good enough outcome” (2009, p. 141) thus deprives the patient and in my mind becomes part of the betrayal. This is a betrayal of the hopes and expectations of work that can never be fulfilled. This becomes more complex for the trainee with professional aspirations.

Trainees find themselves in an exceptional position as they are simultaneously within an analysis whilst also seeing patients and learning the craft of analysis. Trainees are there because they share an inner commitment to the work and the modality and so they are motivated to keep on going. This is tough. Trainees are told to ‘stay with it’, unerringly, and there is faith in the process. The strong commitment and deep psychological work must provide some foundation of resilience. Yet what happens when the ‘staying with it' becomes genuinely harmful? How do we know that the experience of being in the alchemical *vas* has become truly rotten rather than a gradual fermenting process?

The total destruction of the analytic relationship means there will be no *coniunctio*, no union or sacred marriage, or quaternity: no one, no two, not three, never to reach four. Where in the alchemist’s tome is the passage showing what happens when the cycle is incomplete? When the trainee stands outside of the *vas* without an analyst, what is left to do except admit defeat? It is not poetry. It has become a stolen process, a broken promise, one that is antithetical of containment.

## Turbulence on the Horizontal Plane

### Sibling Love and Hate

I am adding the sibling relationship to this alchemical mix and thinking of love and hate, of sibling rivalry, and of re‐enactment in the analytic rupture. Siblings often come in multiples. But how many siblings is not of the child’s choosing. Mitchell in describing the arrival of a new baby into the household says, “The shock of the sibling trauma will also be repeated and have to be reworked through in any future event that displaces and dislodges a person from who and where they thought they were” (2003, p. 205). I suggest that something of this shock reverberates for a trainee who discovers they share an analyst with a peer. There is a gentle rocking of the analytic containment— ‘How is this possible when it was always just the two of us?’.

There has been a tendency in psychoanalysis when considering siblings to focus on rivalry for the parent (Bank and Kahn, 1982; Coles, 2003/2018). Stephen Bank and Michael Kahn (1982) instead decided to look directly at siblings’ relationships. They make a valuable contribution, in what was a neglected area of psychology, researching siblings and their shared emotional connections: “We had been taught that siblings are, at best, minor actors on the stage of human development, that their influence is supposed to be fleeting, and that it is the parents who principally determine one’s identity” (Bank and Kahn, 1982, p. 5). Yet as the research indicates, the sibling bond is “one of life’s most enduring and influential relationships” (1982, p. 4) and with a “different texture” than those between married couples, and children and parents (1982, p. 4). The story is one of enmeshment, dispute, ambivalence, admiration, deep dependence; the origins of which can lie in early childhood. Older siblings can take on the role of attachment figures, and transitional relationships can form between siblings. Siblings can show and express deep care and loyalty for each other, and strong bonds can develop particularly with a shared traumatic upbringing (Bank and Kahn, 1982). Furthermore, siblings can be seen to play a positive role on each other’s development, as well as a long‐term impact on development of the self (Bank and Kahn, 1982; Tickell, 2024; Wittenberg, 2009). Bank and Kahn’s table (1982) identifies major patterns in sibling relationships showing a ‘Fused’ type of relationship identified as ‘Lack of self’ at one end: “We’re just like each other. There is no difference.”; and a ‘Disowned’ type of relationship identified as ‘Estrangement’ at the other: “We’re totally different from one another. I don’t need you, I don’t like you, and I don’t care if I never see you again.” (Table 4.1, Bank & Kahn, 1982, p. 85).

The unique bond shared with siblings is reflected and at times enacted through relationships with peers, partners, colleagues and friends (Bank and Kahn 1982; Coles 2003/2018; Tickell 2024). These sibling dynamics were highlighted in Sophia Tickell’s exploration of sibling dynamics on practitioners and in group work in trainings: “What was actually happening for me in practice … the big influence was the sibling dynamic” (Tickell, 2024, p. 222) and *all* her participants identified ‘sibling dynamics in group work during training’ (Tickell, 2024, p. 223).

Mitchell has written extensively on siblings and made a large contribution in bringing the “missing siblings” (2003, p. 32) to the forefront of psychoanalytic thinking:

We completely forget that Oedipus and his children are brothers and sisters. I want to take this as a metaphor for what I see as the suppression of the significance of laterality —of siblings, and their successors, peers and affines—from a psychoanalytic understanding of the construction of psychic life. (Mitchell, 2003, p. 32–33)

She opened up the lateral dimension of siblings from the narrow focus of the vertical perspective of parent‐child—a place where “siblings have been cast as hated rivals for the mother’s breast” (Vivona, 2007, p. 1192)—to one in which the psychic life of sibling relationships is viewed with equal importance. In understanding the unique challenges that siblings face: the “fundamental desire to murder your sibling” (Mitchell, 2003, p. 35) and how “violence must be turned into love” (2003, p. 35), Mitchell recognises the trauma of displacement (Mitchell, 2003). Taking hold of this horizontal dimension, Vivona (2007) suggested that a child might use differentiation with its sibling as a way of managing rivalry, in an “active, unconscious process of identity development” (Vivona, 2007, p. 1198), as well as a way of uniting opposites.

Writing on the importance of sibling relationships, Prophecy Coles (2003/2018) argues that Mitchell’s view of the catastrophic nature of the birth of a new sibling does not allow for relationships that “can open up the world in a new and positive way” (Coles, 2003/2018, p. 88). She wants to “resist the tendency to reduce them to hated, unwanted, and distorted figures to our developing selves” (2003/2018, p. 89) and emphasises the positive nature of sibling interaction. The concept of ‘brotherly love’ is central in understanding the deep affection, loyalty and friendship that develop out of our childhood ties. Coles’ rethinking of Freud and Dann’s 1951 paper 1 on the six war‐orphaned children (who had no experience of parents) suggests the children showed a capacity “to draw psychic nourishment and strength from an identification with the group” (Coles, 2003/2018, p. 77).

### When Trainees Share an Analyst

I question whether (and how) all these invisible threads, these unconscious connections play out through trainings. It is interesting how trainings call trainees (who share an analyst) *siblings*. What is this setting up in the unconscious for the new trainees embarking with peers on a long analytic training? I think this tension is not simply about siblings who share an analyst—although perhaps this holds the most tension—but peer siblings who share the training, unknown peer siblings in the training, or those from another training, known and unknown. My experience is that these connections can create ripples from the outset. Mary Addenbrooke (1997) reflects on her own personal illustration of regression within a training:

I often landed myself in scrapes by making statements too dramatically, and felt like a blundering elephant by comparison. There is little doubt that this replicates my jealousy of my sister who was so much more charming and gracious than I. My fury and envy were an uncomfortable burden to me as a child and one which I was at pains to keep as well concealed as I could. (Addenbrooke, 1997, p. 514)

She recounts an incident of watching a young girl playing, tossing her large teddy bear into the air, only ever directly throwing it to her brother, yet it being caught and thrown by other children. Play is developing, then moments when unable to tolerate the separation from her bear, the other children sensing this and throwing the bear back to her, before she reclaims it with a gentle hug and the bear is launched out again. Addenbrooke discusses the vignette as how we share parts of ourselves in a group “and how risky it feels to let other people in the group see, let alone throw in the air, what you bring”. She claims the teddy bear game as a metaphor for how she and her fellow trainee group played together and how “play is trying things out” (Addenbrooke, 1997, p. 516).

What does sharing the *analyst*‐bear look like? How might the analyst‐bear be suddenly flung out to the group in a revelatory display? The story begins with ‘My analyst …’. There is rarely a name. There is often reverence, sometimes a joke but the frame belongs to the patient‐narrator. The patient‐narrator is in control of the analyst‐bear throw. Trainees tend to listen hard and may respond with their own analyst‐bear tale, and some may never share, hugging the analyst‐bear close, not ready to give up the transitional object to the group. But mostly the sharing is a bonding experience. At least, on the surface.

I once responded to a peer with whom I was on an analytic training that my analyst (unlike hers) did not usually say hello—he would greet me with a kind of silent nod and smile but there was no verbal communication. The woman looked aghast and said that *her* analyst would never do that to her, and she made it clear that this was somehow a huge failing on *my* analyst’s part. We later discovered that we shared the same analyst. My analyst is not the same as your analyst, even when they are! The analyst’s shape‐shifting guise is a further testament to the individuality of the alchemical *vas*. My analyst *cannot be* the same as your analyst, even when they are.

However, it seems to underline the symbolic nature of psychoanalysis and the wholly individual analyst–patient relationships that take place in their own sphere, separate and discrete from each other. It emphasises the duality of the relationship and makes me consider this notion that *more than two* might well put too much pressure on the alchemical woodcut process. From the immersion in the bath, and *coniunctio*, to the death and ascent of the soul and onwards, it is a unique, celestial tale full of personal elephant blunderings and hellos and not‐hellos, which form a one‐off concoction between the analyst‐patient couple. The *vas* contains the matters of psyche whilst they cook and coagulate on the inside, but it cannot always protect from judgements and breaches entering from the outside.

It is apparent how sibling dynamics may influence the powerful unconscious processes that are set up in a long training and analysis between trainees and the shared analyst. I would suggest that it is possible, if not probable, that when a trainee in analysis finds themselves with a new set of designated peers and siblings, that this can open an intense array of responses for the trainee, in terms of patterns of identification, and that these can be transferred onto the new relationships. Each trainee is bringing their own sibling experience into the mix. Analytically speaking this is not unexpected and may, overall, be navigated with minor strain or fuss.

Donald Winnicott describes in *The Piggle* his young patient’s very evident struggle for identity after the birth of her younger sister:

She becomes easily bored and depressed which was not evident before, and is suddenly very conscious of her relationships and especially of her identity. The acute distress, and the overt jealousy of her sister, did not last long, though the distress was very acute. (Winnicott, 1977/1991, p. 6)

The Piggle is experiencing the disruption of becoming “no one” (Mitchell, 2003, p. 29) as the new baby enters the frame. A mourning takes place by the child for its “unique self” (Mitchell, 2003, p. 30) as they wrestle with the overwhelming sense of identity loss. Mitchell describes the displacement of an older child who becomes “not just displaced, but for a time is without a place – someone else is what she is” (Mitchell, 2003, p. 47). I imagine this movement from someone to no one, and from place to no place could be part of what is happening in the complex pattern of identification and relationships for trainees on a training.

Patterns in sibling behaviour are observable in even pre‐natal children. Piontelli’s research using ultrasound scans demonstrated the very early life between twins noting the gentle strokes of ‘the kind twins’ who shared a womb, as well as the ‘reactive’ kicks between twins who fought right through the pregnancy: “Jesus … it is kicking and punching the other one! … look! … now the right one has reacted too … pushing it back with its knees … it doesn’t want its space to be occupied …” (Piontelli, 1992, p. 150). Post‐natal observations noted the intense rivalry and jealousy between the ‘reactive’ twins who found it difficult to share and not have their separate space. They instigated separate drawers in their shared bedroom leading the mother to comment: “woe betide me if I exchange their clothes!…” (Piontelli, 1992, p. 160). It was suggested in the study that they “received just a ‘half’ of what they feel they could have had otherwise”. How we occupy space is very much the focus of individuals within an analysis, of trainees within a cohort, and particularly of trainees sharing an analyst. Siblings can also show great resilience, as seen with Delia (a less favoured twin) who is good natured and makes use of other people, teachers and friends, in order to gain the attention and interest she needs (Piontelli, 1992).

These early experiences of sibling dynamics—the kindness, rivalry, independence, hatefulness and cooperation—are visible in our training groups, when we reach out to give a gentle stroke to a peer or react with a hefty kick (metaphorically speaking). These training dynamics become part of what is held in the alchemical *vas* in the analysis. However, as I stated, ‘experiences of boundary violation within training risk weakening the capacity of the training womb‐*vas*, for the peers that share the analyst, that might result in internal chaos’. The analyst who makes this choice has lost the capacity to work analytically. The countertransference illusion becomes displaced into everyone else. The ‘separate drawers’ become muddled, and those trainees who share the training‐womb *vas* experience the madness of being in dissolution without knowing why.

### Dreaming in *Nigredo*

I had a number of dreams towards the end of my analysis, one after another, nightmare scenarios. They were about houses on fire, someone killing off the passengers on a train, my terror of being attacked upon seeing a man in a graveyard, taking a thorn out of my foot, losing my cat, feeling fearful in my house, my need to move as something bad was going to happen, preparing for the end of the world, being kept in the dark and finding a hidden monitor, being in New York when a hostile takeover unfolds, terror and running for my life, barbarians killing people, driving over a cliff and realising it was real and not a dream, dreaming of my other training siblings who shared the analyst, being inside a car with wolves inside and out, throwing women out of my house. In the last dream, I was terrified of the man who was going to rape me in a house with walls blistered by fire. These dreams stopped the day after I heard my analyst had been suspended. I, or my unconscious, was working hard, trying to make sense of something. This is the madness of being in dissolution without knowing why. Looking back, I can see I already knew the work was no longer offering containment.

## The Training Complex

My hypothesis involves thinking about the unconscious sideways (lateral) transmission of material (data, trauma) through the ‘analytic family’ and how individuals position themselves in a powerful enactment that I will call ‘the training complex’. I suggest how an unconscious sideways transmission of material between peers who share an analyst and the analyst in intensive training might involve sibling rivalry; enactments within the frame, such as elevating one patient (the golden child) whilst putting down another; power dynamics between siblings; shared dreams between siblings; and unknown decrees like ‘I knew I couldn’t speak about her’ (feeling unable to mention one’s sibling in the analysis).

I suggest that this complex contains a multitude of relationship threads, i.e. conscious to conscious, conscious to unconscious, and unconscious to unconscious, both vertical and horizontal dynamics, as indicated in Jung’s diagram (see Figure 2, Jung, 1946, para. 422).

**Figure 2:**
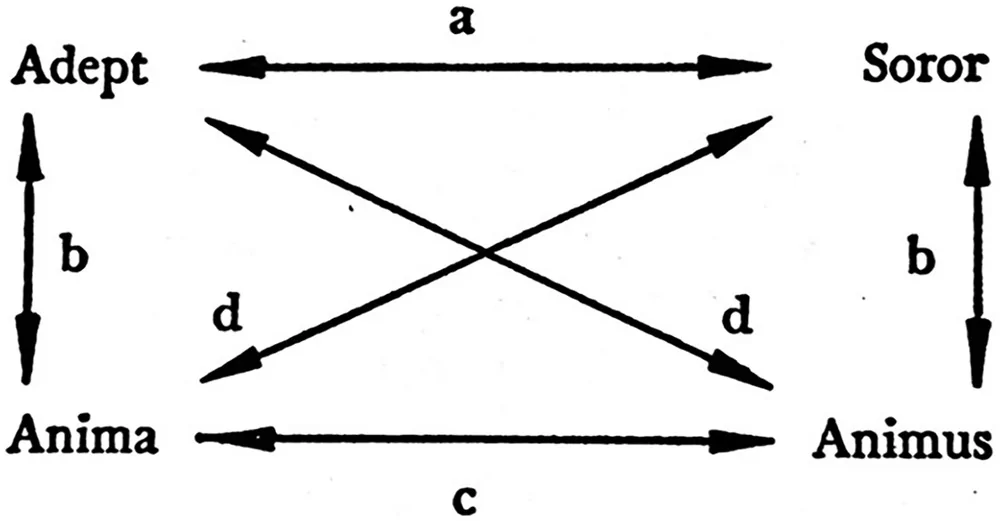
Jung’s “counter‐crossing transference relationship.” *Source:* C. G. Jung (1946/1966), *The Psychology of the Transference*, in *Collected Works*, Vol. 16, ¶422. Reproduced with permission of Princeton University Press.

These threads cross between analyst, peer siblings, and peers, and into the wider organisation. Any ensuing rupture explodes what was symbolic into a catastrophic family melee, disrobing the analyst, and igniting a very real and painful crisis for those who are caught up in it. These are the psychic wounds of Jung’s complexes. Jung recognised complexes as being “splinter psyches” …. “The aetiology of their origin is frequently a so‐called trauma, an emotional shock or some such thing, that splits off a bit of the psyche” (Jung, 1948, para. 204). The emotional shock within the training body is congruent with the felt disturbance of dozens of splinter psyches, made conscious through years of individual and intensive work constellating in a group expression of crisis. This reflects an element of contagion within the training complex.

However, if one also looks at the training organisation as a whole body or organism, then the destructiveness of the impact might also cause some individuals to split off, dissociate, shut down from the group in an attempt to keep themselves safe from harm, thus causing splinter psyches within the training organisation. For the profession, the ethical boundary violation can be like the “skeleton in the cupboard” of whom Jung says, “the existence of which must not be mentioned in their presence on pain of death, so great is their fear of the lurking spectre” (Jung, 1948, para. 208). Then the training complex becomes part of the phenomenon of silencing which can weaken and undermine processes, leading to rupture within the institute (Sinsheimer, 2014, p. 147).

I also consider the destructive nature of the experience of boundary violation on the individual and on the training organisation to be an amplification of the “psychic disintegration” (Jung, 1948, para. 202), the “violent explosion of affect” (Jung, 1921/1971, para. 469) captured by Jung in his description of complexes. The destructiveness can ricochet across the whole organisation and be felt in many ways.

I do not think the area of trainees undergoing a training with a shared analyst has been researched sufficiently. It is expected and assumed that this sharing happens. I suggest the single container of analyst and patient can become contaminated through the multiple containers which are being held by one analyst, and as the siblings may have a separate relationship to each other within the training, this is also very unusual. In most circumstances a therapist will not take on a patient who has a known relationship with another patient as it can confuse or muddy the transference. I think how analysts give careful consideration to patients not coming into contact with each other—on the doorstep, in the waiting room, outside the premises—and yet training organisations knowingly enrol new trainees with the same analyst who will be working alongside each other for some years. This is particularly the case as candidates must usually work with an analyst from their own training organisation. There are potentially repercussions for what this sets up within the transference. Jung says, “We do certain things simply without thinking, because they have always been done like that” (Jung, 1946, para. 437) on discussion of the form of sister‐brother marriages.

I want to expand on this thinking and suggest that if the analyst is holding a multiple set of sibling containers then perhaps along with Jung’s: “confronting one another in enmity or attracting one another in love” (Jung, 1956, para. 1) and Samuel’s: “pervasive intermingling of the two personalities” (Samuels, 2006, p. 186) then I can imagine this analytic family container heating up with an intense mix‐up of primitive sibling rivalries. I suggest this powerful blend of incestuous and murderous sibling desires (Mitchell, 2003) might impact the containment of the individual analyses. It may simply not always be possible to withstand the pressure.

John Byng‐Hall suggests “marriages are based on finding partners who can play out some of the parts or counterparts in each others [*sic*] scripts” (Byng‐Hall, 1985, p. 301). I speculate how family scripts might play out through the analytic family. Scripts can also be rewritten. Shakespeare propelled sibling rivalry to extraordinary heights in the tragedy of *King Lear*. King Lear—in asking his daughters a simple question, ‘Which of you shall we say doth love us most …’ (Shakespeare, 1608/2024, p. 161)—is breaking open the *prima materia* of his inflated narcissistic ego with dire consequences. It so shocked audiences at the time, who were used to the fairy‐tale *good ending*, that it was not often performed (see Figure 3).

**Figure 3:**
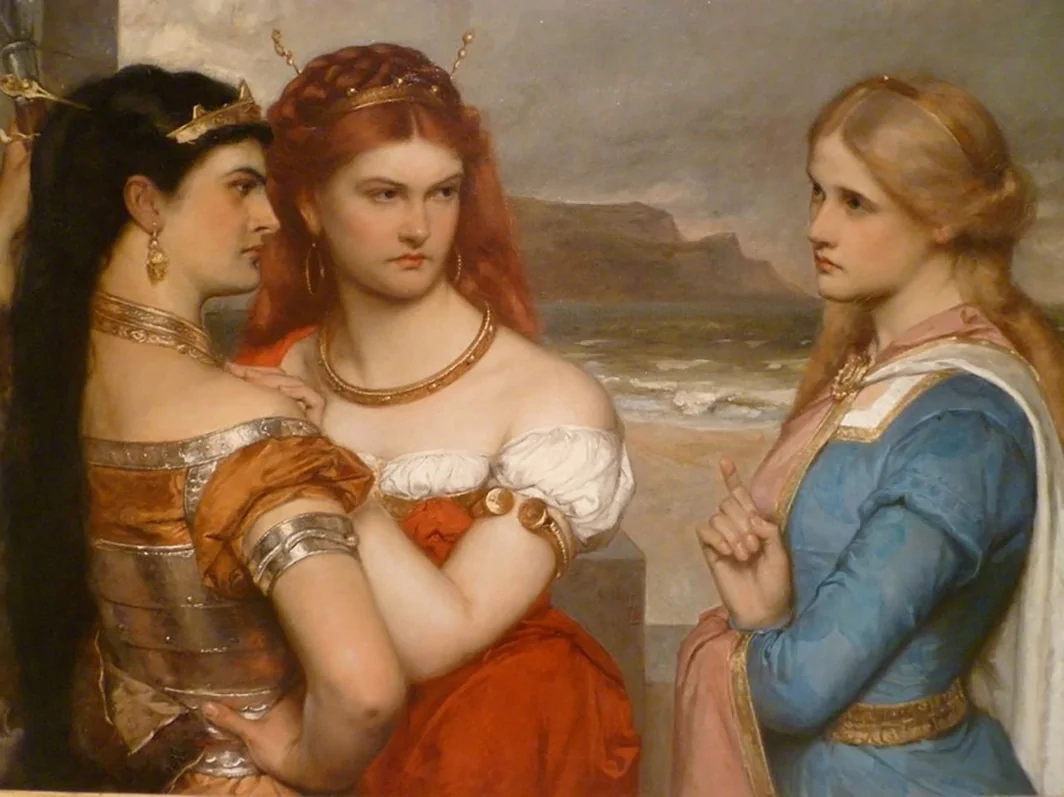
*The three daughters of King Lear* (Pope, 1875). Oil on canvas, Museo de Arte de Ponce, Puerto Rico. Public domain.

### Siblings as opposites—finding equilibrium

In my own experience, the story goes that the day I was born my new and older brother of 19 months ran up and down the stairs being sick. It was a home birth. This family story encapsulates the rupture within the family container due to my entrance. The family folklore was permissible because we survived this upset, and he had not tried to kill me. An equilibrium was established.

In *Mysterium Coniunctionis*, “The factors which come together in the coniunctio are conceived as opposites, either confronting one another in enmity or attracting one another in love” (Jung, 1956, para. 1). Is this not a description of the rivalry of siblings? Mitchell focuses on murderous desires turning to love and Coles on the natural strength of love portrayed by siblings. They are showing opposite experiences of siblings. Bank and Kahn’s table (1982, p. 85, Table 4.1) shows the ‘major patterns of identification and relationship between siblings’ and gives an example of spectrum—from siblings fused together at one end whilst disowned at the other.

I conclude that sibling dynamics are symbolic of the opposites we hold within us: they represent something of the fusion and something of the disowned and this is reflected in the turbulence of the broken container. Edinger believes that to know the opposites is to understand the psyche, but it is a dangerous endeavour:

It’s *the* basic drama that goes on in the collective psyche. Every war, every contest between groups, every dispute between political factions, every game, is an expression of coniunctio energies. Whenever we fall into an identification with one of a pair of warring opposites, we then lose the possibility, for the time being anyway, of being a carrier of the opposites. And instead we become one of God’s millstones that grinds out fate. At such times one still locates the enemy on the outside and in so doing is simply a particle. (Edinger, 1994, p. 15)

It is symptomatic of the way an organisation is left holding all the broken pieces and how the voices within the organisation move between these different pieces as they try to put something back together. The voices can become warring opposites, trying to fix something that cannot be fixed, because the analyst who carried the heavy responsibility has passed it over like a baton and left the scene. How do we not become God’s millstone that grinds out fate? How do we come together at this time to put things back together?

## Conclusion

It is difficult to grasp the behaviours and thinking that lead to boundary violations, but equally difficult to know how to deal with the aftermath. I consider Kirsch’s reflection on the “incomprehensible blindness” (Kirsch, 2021, p. 223), and Gabbard’s tireless writing on the impact of sexual boundary violation in the analysis and within the training organisation, and the sense of “growing pessimism” (2017, p. 151) on turning the tide of analyst–patient relationships. The disruption can greatly affect the training body and organisation which can lead to internal changes within the training institutes after an ethical breach as well as a need to understand the trauma on a personal and collective level (Anonymous, 2005; Burka, 2014; Burka et al., 2019; Fromberg, 2014; Kirsch, 2021; Levin, 2014; Sinsheimer, 2014; Wallace, 2014; Young, 2014). These writings address just some of the disruptions that follow such enactments. Part of my writing this paper has been to find a way of thinking about something that I have discovered is incredibly hard to think about. Thoughts are constantly jamming—reinterpreted, ignored, split off, forgotten, reframed—because my reality was violated. This has been about regaining my own capacity to think after the event.

I see Sisyphus endlessly pushing a rock up a hill; or the stuck part of an analysis where the power of the third area cannot be sustained, and so the rock rolls back, but within an organisation something of this Sisyphean task must be held (that maybe cannot be held by the analyst who is caught up in harmful reenactments or who feels the binding pressure of containment). And then the group must discover for themselves how to move the rock. In this way I suggest that the training complex—with its powerful and destructive enactments—must be worked through organisationally.

## Footnotes

^1^
Freud, A., & Dann, S. (1951). An experiment in upbringing. *Psychoanalytic Study of the Child*, 6: 127–168.
